# Reading the Freudian theory of sexual drives from a functional neuroimaging perspective

**DOI:** 10.3389/fnhum.2014.00157

**Published:** 2014-03-18

**Authors:** Serge Stoléru

**Affiliations:** ^1^Institut National de la Santé et de la Recherche Médicale, INSERM U669Villejuif, France; ^2^Département Biologie, Médecine et Santé, Université Paris-Decartes, UMR-S 669Paris, France

**Keywords:** sexual drives, psychoanalysis, functional neuroimaging, sexual arousal, motivation, neurophenomenology, neuropsychoanalysis

## Abstract

One of the essential tasks of neuropsychoanalysis is to investigate the neural correlates of sexual drives. Here, we consider the four defining characteristics of sexual drives as delineated by Freud: their pressure, aim, object, and source. We systematically examine the relations between these characteristics and the four-component neurophenomenological model that we have proposed based on functional neuroimaging studies, which comprises a cognitive, a motivational, an emotional and an autonomic/neuroendocrine component. Functional neuroimaging studies of sexual arousal (SA) have thrown a new light on the four fundamental characteristics of sexual drives by identifying their potential neural correlates. While these studies are essentially consistent with the Freudian model of drives, the main difference emerging between the functional neuroimaging perspective on sexual drives and the Freudian theory relates to the source of drives. From a functional neuroimaging perspective, sources of sexual drives, conceived by psychoanalysis as processes of excitation occurring in a peripheral organ, do not seem, at least in adult subjects, to be an essential part of the determinants of SA. It is rather the central processing of visual or genital stimuli that gives to these stimuli their sexually arousing and sexually pleasurable character. Finally, based on functional neuroimaging results, some possible improvements to the psychoanalytic theory of sexual drives are suggested.

According to Freud, the concept of sexual drive is a defining element of psychoanalysis. However, in a footnote added in 1924 to his “Three Essays on the Theory of Sexuality” (Freud, 1905), he wrote: “The theory of the instincts is the most important but at the same time the least complete portion of psychoanalytic theory”. The theory of sexuality elaborated by Freud was among the reasons why psychoanalysis met so much resistance, not only from the patients, but also from the scientific community. In his 1920 preface to the fourth edition of “Three Essays on the Theory of Sexuality”, Freud wrote: “[…] it is satisfactory to be able to record the fact that interest in psycho-analytic research remains unimpaired in the world at large. […] That part of the theory, however, which lies on the frontiers of biology […] is still faced with undiminished contradiction”.

Because sexuality is so important in psychoanalytic theory, because the instincts are “the most obscure element of psychological research” (Freud, 1920), because it has been so controversial, because that part of the theory lies on the frontier between the mental and the somatic (Freud, 1915a), and because neuropsychoanalysis endeavors to find the neural underpinnings of psychoanalytic concepts, it appears to me that one of the chief tasks of neuropsychoanalysis is to investigate the neural correlates of sexual drives. In 1996, our group embarked on this task using functional neuroimaging techniques, initially Positron Emission Tomography (PET), then functional Magnetic Resonance Imaging (fMRI). Since that time, more than 70 articles have reported studies of human sexual desire/arousal based on functional neuroimaging techniques, whether in healthy or in pathological samples (Stoléru et al., 2012). Thus, now is the time to return to Freud’s original writings and try to assess how findings of functional neuroimaging studies relate with his theory of sexual excitement and sexual drives. Are the results of functional neuroimaging experiments consistent with the Freudian model of sexual drives? Are they consistent only in some respects? Can modern studies actually help psychoanalysis to reformulate certain aspects of this model? Those questions are among the points examined hereunder.

Freud was quite open to such a re-examination of his theory: “The deficiencies in our description would probably vanish if we were already in a position to replace the psychological terms by physiological or chemical ones. […] Biology is truly a land of unlimited possibilities. We may expect it to give us the most surprising information and we cannot guess what answers it will return in a few dozen years to the questions we have put to it” (Freud, 1920). Indeed, this projection into the future was echoed a few dozen years later by Kandel when he cogently spelled out an agenda for psychoanalysis and neurobiology to engage in a dialogue, including regarding the understanding of sexual drives (Kandel, 1999).

## An outline of Freud’s theory of sexual excitement and sexual drives

We shall first consider Freud’s account of sexual excitement, mainly on the basis of “Three Essays on the Theory of Sexuality” (Freud, 1905). Then, we shall focus on his theory of sexual drives, mainly based on “Instincts and their Vicissitudes” (Freud, 1915a). When he started elaborating his theory of sexual excitement, Freud was focusing on a phenomenon that is, at least in part, directly observable, including genital, cardiovascular and respiratory manifestations. By contrast, a sexual drive cannot be directly observed; it is a construct inferred from psychoanalytic (or other) investigation with an aim to explain various phenomena, in particular sexual excitement. “You never experience a drive directly. […] The feeling of thirst is not itself a drive, which is not something you experience; rather, the concept of thirst explains why you feel thirst when you need water” (Solms and Zellner, 2012). Thus, from an epistemological viewpoint, there is a sharp distinction between the concepts of sexual excitement and of sexual drives.

Regarding sexual excitement, Freud wrote: “This apparatus [i.e., the somatic and psychical sexual apparatus] is to be set in motion by stimuli, and observation shows us that stimuli can impinge on it from three directions: from the external world by means of the excitation of the erotogenic zones […], from the organic interior by ways which we have still to explore, and from mental life, which is itself a storehouse for external impressions and a receiving-post for internal excitations. All three kinds of stimuli produce the same effect, namely a condition described as “sexual excitement”, which shows itself by two sorts of indication, mental and somatic” (Freud, 1905).

Elaborating on the feeling of tension, Freud further described the peculiar mixture of unpleasure and pleasure associated with sexual excitement: “I must insist that a feeling of tension necessarily involves unpleasure. What seems to me decisive is the fact that a feeling of this kind is accompanied by an impulsion to make a change in the psychological situation, that it operates in an urgent way which is wholly alien to the nature of the feeling of pleasure. If, however, the tension of sexual excitement is counted as an un-pleasurable feeling, we are at once brought up against the fact that it is also undoubtedly felt as pleasurable. […] How, then, are this unpleasurable tension and this feeling of pleasure to be reconciled?” (Freud, 1905). Could it be that, in order to motivate human beings to advance from low to high excitement and ultimately to orgasm, two incentives operate, i.e., (i) the pleasure of excitement, a pleasure that grows as excitement increases; and (ii) the tension which the individual will work to ease, thereby obtaining additional pleasure.

One of the essential tenets of the Freudian theory of sexual excitement is the concept of erotogenic zones: “[…] excitations of two kinds arise from the somatic organs, based upon differences of a chemical nature. One of these kinds of excitation we describe as being specifically sexual, and we speak of the organ concerned as the “erotogenic zone” of the sexual component instinct arising from it. […] in scopophilia and exhibitionism the eye corresponds to an erotogenic zone” (Freud, 1905). As shown below, in most functional neuroimaging studies of sexual excitement, investigators have used visual sexual stimuli (VSS), thus relying on scopophilic tendencies of both healthy subjects and patients to induce sexual excitement.

Not only did Freud elaborate a theory of sexual excitement, but he also proposed a theory of its inhibition. The latency period, that extends approximately from 6–7 to 10–11 years of age, has a great importance in this regard: “It is during this period of total or only partial latency that are built up the mental forces which are later to impede the course of the sexual instinct and, like dams, restrict its flow—disgust, feelings of shame and the claims of aesthetic and moral ideals” (Freud, 1905). We shall see later that the neural model of sexual arousal (SA) also comprises inhibitory components.

Regarding Freud’s theory of sexual instincts/drives, in the first lines of “Instincts and their vicissitudes” (Freud, 1915a), he acknowledged that the very existence of instincts was postulated rather than demonstrated: “We have often heard it maintained that sciences should be built up on clear and sharply defined basic concepts. In actual fact no science, not even the most exact, begins with such definitions. The true beginning of scientific activity consists rather in describing phenomena and then in proceeding to group, classify and correlate them. Even at the stage of description it is not possible to avoid applying certain abstract ideas to the material in hand, ideas derived from somewhere or other but certainly not from the new observations alone. Such ideas—which will later become the basic concepts of the science—are still more indispensable as the material is further worked over. They must at first necessarily possess some degree of indefiniteness; there can be no question of any clear delimitation of their content. So long as they remain in this condition, we come to an understanding about their meaning by making repeated references to the material of observation from which they appear to have been derived, but upon which, in fact, they have been imposed. Thus, strictly speaking, they are in the nature of conventions—although everything depends on their not being arbitrarily chosen but determined by their having significant relations to the empirical material, relations that we seem to sense before we can clearly recognize and demonstrate them”. Then, he added a few lines below: “A conventional basic concept of this kind, which at the moment is still somewhat obscure but which is indispensable to us in psychology, is that of an “instinct””.

In the “Three Essays on the theory of sexuality”, Freud had already given what he had called a “provisional” definition of instincts: “By an “instinct” is provisionally to be understood the psychical representative of an endosomatic, continuously flowing source of stimulation, as contrasted with a “stimulus”, which is set up by single excitations coming from without. The concept of instinct is thus one of those lying on the frontier between the mental and the physical. […] What distinguishes the instincts from one another and endows them with specific qualities is their relation to their somatic sources and to their aims. The source of an instinct is a process of excitation occurring in an organ and the immediate aim of the instinct lies in the removal of this organic stimulus”.

Freud described four crucial defining characteristics of sexual drives (1915a). Firstly, “By the pressure of an instinct we understand its motor factor, the amount of force or the measure of the demand for work which it represents. The characteristic of exercising pressure is common to all instincts; it is in fact their very essence. […] if we speak loosely of passive instincts, we can only mean instincts whose aim is passive”.

Secondly, “The aim of an instinct is in every instance satisfaction, which can only be obtained by removing the state of stimulation at the source of the instinct. But although the ultimate aim of each instinct remains unchangeable, there may yet be different paths leading to the same ultimate aim; so that an instinct may be found to have various nearer or intermediate aims, which are combined or interchanged with one another”. In keeping with this notion of intermediate aims, in later passages of “Instincts and their vicissitudes”, Freud uses the term “aim” to refer, not to the satisfaction of instincts, but to the actions performed by the subject to reach satisfaction, e.g., to torture in the case of sadism, to watch in the case of scopophilia.

Thirdly, “The object of an instinct is the thing in regard to which or through which the instinct is able to achieve its aim. It is what is most variable about an instinct and is not originally connected with it, but becomes assigned to it only in consequence of being peculiarly fitted to make satisfaction possible. The object is not necessarily something extraneous: it may equally well be a part of the subject’s own body”.

Finally, “By the source of an instinct is meant the somatic process which occurs in an organ or part of the body and whose stimulus is represented in mental life by an instinct. We do not know whether this process is invariably of a chemical nature or whether it may also correspond to the release of other, e.g., mechanical, forces. The study of the sources of instincts lies outside the scope of psychology. Although instincts are wholly determined by their origin in a somatic source, in mental life we know them only by their aims. An exact knowledge of the sources of an instinct is not invariably necessary for purposes of psychological investigation; sometimes its source may be inferred from its aim”.

The importance of instincts in psychoanalytic theory, their still obscure character combined with the new opportunities to study SA offered by neuroimaging techniques starting in the nineteen nineties make of the instincts one of the objects “par excellence” of present and future neuropsychoanalytic investigation. Thus, the neuropsychoanalytic investigation of sexual instincts is motivated and made possible by the following conjunction: (i) in Freud’s own view, what makes the psychoanalytic theory of instincts particularly difficult to elaborate is the complex—both representational and somatic—nature of sexual instincts; and (ii) since a few years, the availability of functional neuroimaging techniques might shed light on the somatic, at least on the cerebral, aspects of sexual instincts.

## A neurophenomenological model of sexual arousal

The neurophenomenological model of SA^1^ proposed here has been essentially derived from functional neuroimaging studies of our group (e.g., Redouté et al., 2000; Stoléru et al., 2003; Stoléru, 2008) and from the meta-analysis of similar studies by other teams (Stoléru et al., 2012). These studies aim to identify the brain regions that show a response to sexual stimuli and then to elaborate a theoretical model of SA. Most of the responses observed are an increased activity (activation), but responses can also consist in a decreased activity—a deactivation. The identification of the regions responding to sexual stimuli can provide insights into the cerebral basis of SA, especially when it is combined with previous knowledge on the function of those areas and on the phenomenology of SA. The term “neurophenomenological” is meant to reflect the attempt of the model to trace the connections between the neural and the experiential responses to sexual stimuli.

## Methods

The stimuli used in these experiments can in principle be external stimuli, but also internal stimuli, i.e., fantasies. So far, nearly all experiments have used external stimuli, most commonly visual ones. While no study has used internal stimuli (sexual fantasies), in a few studies, SA was induced by audiovisual, or verbal, or olfactory stimuli, or by masturbation by partner (Stoléru et al., 2012). Thus, hereafter we describe the experimental paradigm based on VSS. Subjects are studied in various experimental conditions and their brain responses are compared across these conditions. Conditions are defined by the type of visual stimuli presented to participants. In a typical study, in the sexual arousal condition (SA) subjects view sexually explicit photographs or film clips. In the neutral condition (N), subjects are presented with sexually neutral photographs or film clips. In some studies, a third condition is used to show the specifically sexual nature of the arousal induced by sexual stimuli. For instance, sports videos were presented to demonstrate that potential differences in brain activation between the sexual and the neutral conditions were specifically related to SA and not to any kind of arousal (Arnow et al., 2002). The most frequently used techniques have been fMRI and, less often, PET. Much more rarely, studies have been based on magnetoencephalography, high resolution EEG or Single-Photon Emission Computed Tomography (Stoléru et al., 2012).

SA induced by visual stimuli is assessed through two main approaches: (i) rating scales, presented shortly after the various categories of visual stimuli, to assess levels of perceived SA; and (ii) measurement of erection during the presentation of stimuli through penile plethysmography (also called phallometry). In some studies, authors have used additional measurements, during or immediately after the presentation of stimuli, such as heart rate, respiratory rate and plasma testosterone (e.g., Stoléru et al., 1999; Redouté et al., 2000).

The participants are installed on the bed of the scanner. Typically, a mirror positioned before their eyes reflects a screen located behind their head and the stimuli are presented via a videoprojector. Every effort is made to respect the participants’ privacy throughout the experiment.

### Outline of the neurophenomenological model

The presentation of VSS induces a pattern of cortical and subcortical activation and, to a lesser extent, deactivations (Kühn and Gallinat, 2011; Stoléru et al., 2012; Poeppl et al., 2013; Sescousse et al., 2013). How can these multiple regional brain responses be organized into a phenomenologically meaningful model, i.e., a model that would account for the multiple and various facets comprising the subjective experience of SA? We have proposed a four-component neurophenomenological model, i.e., comprising a cognitive, a motivational, an emotional and an autonomic/neuroendocrine component (Stoléru et al., 1999, 2003, 2008; Redouté et al., 2000, 2005). In addition, each component appears to be controlled by inhibitory processes.

**Figure 1:**
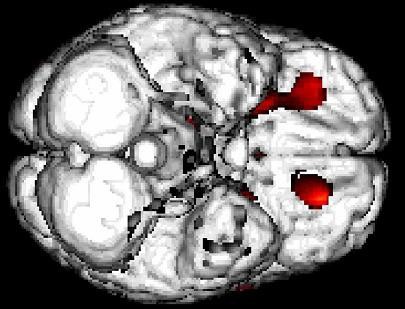
**An inferior view of the brain showing a bilateral activation of the orbitofrontal cortex in response to the presentation of sexually arousing film clips (unpublished figure from study by Redouté et al., 2000)**.

The cognitive component comprises (i) a process of appraisal through which stimuli are qualitatively categorized as sexual incentives and quantitatively evaluated as such; (ii) increased attention to stimuli evaluated as sexual; and (iii) motor imagery whose content is related to sexual behavior. The activation of the orbitofrontal cortex (Figure 1), the fusiform gyri (parts of the inferior temporal cortices), the superior parietal lobules, and of areas belonging to the neural network mediating motor imagery (inferior parietal lobules, left ventral premotor area, right and left supplementary motor areas, cerebellum) are conceived as the neural correlates of the cognitive component. The process of cognitive appraisal of stimuli as sexual is postulated as being the first step in the whole process of unfolding SA, with later processes depending on it.

**Figure 2:**
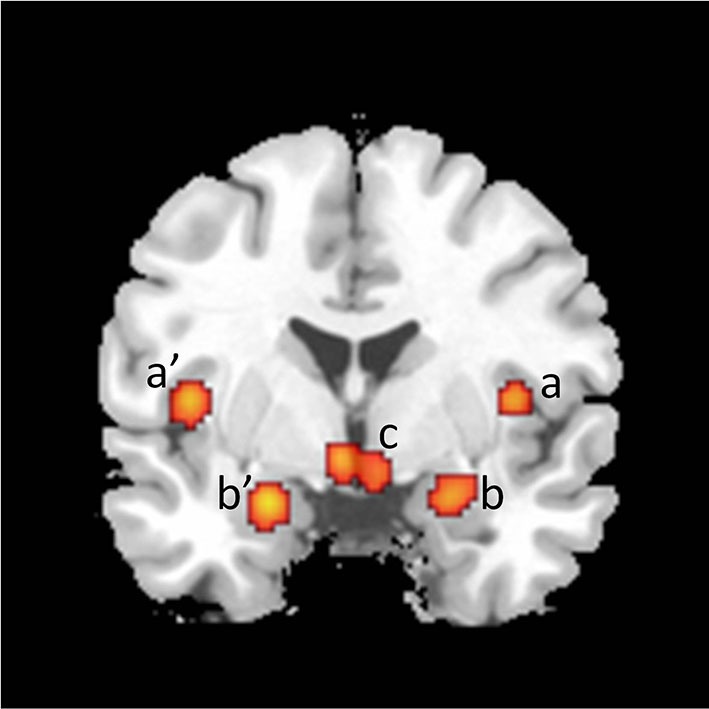
**Coronal section showing activations found in a meta-analysis of 21 studies of brain responses of heterosexual men to sexual stimuli (unpublished figure from study by Stoléru et al., 2012)**. Section is located 1 mm caudal to anterior commissure. a, a’: insula; b, b’: amygdala; c: hypothalamus. Right is to the right.

The emotional component includes the specific hedonic quality of SA, i.e., the pleasure associated with rising arousal and with the perception of specific bodily changes, such as penile tumescence. It also includes other potential emotions associated with SA such as tension, hope, fear, etc. The activations of the primary somatosensory cortex—in the cortical area that receives inputs from the external genitalia—, of the left secondary somatosensory cortex, of the amygdalas, and of the insulas are conceived as neural correlates of the emotional component (Figure 2).

**Figure 3:**
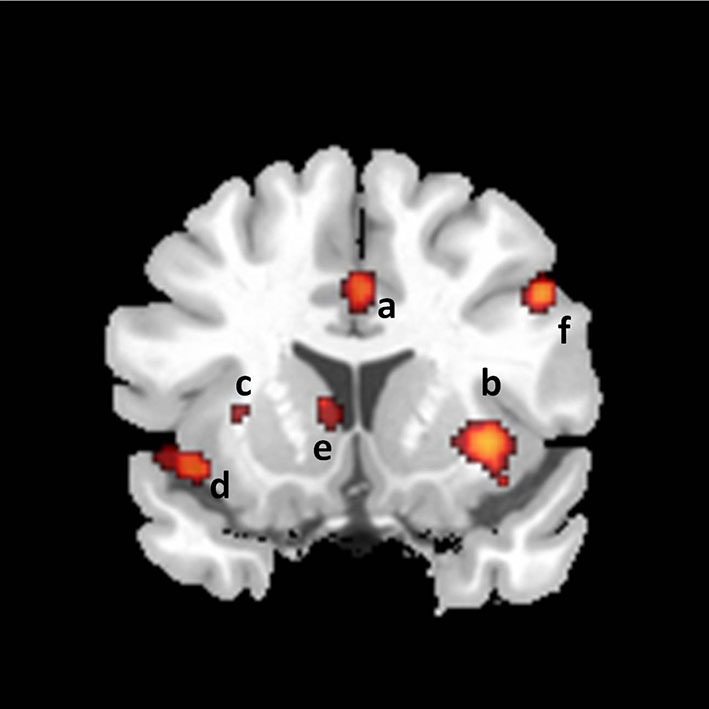
**Coronal section showing activations found in a meta-analysis of 21 studies of brain responses of heterosexual men to sexual stimuli (unpublished figure from study by Stoléru et al., 2012).** Section is located 14 mm rostral to anterior commissure. a: anterior cingulate gyrus; b: claustro-insular cluster; c: claustrum; d: insula; e: caudate nucleus; f: inferior frontal gyrus. Right is to the right.

**Figure 4:**
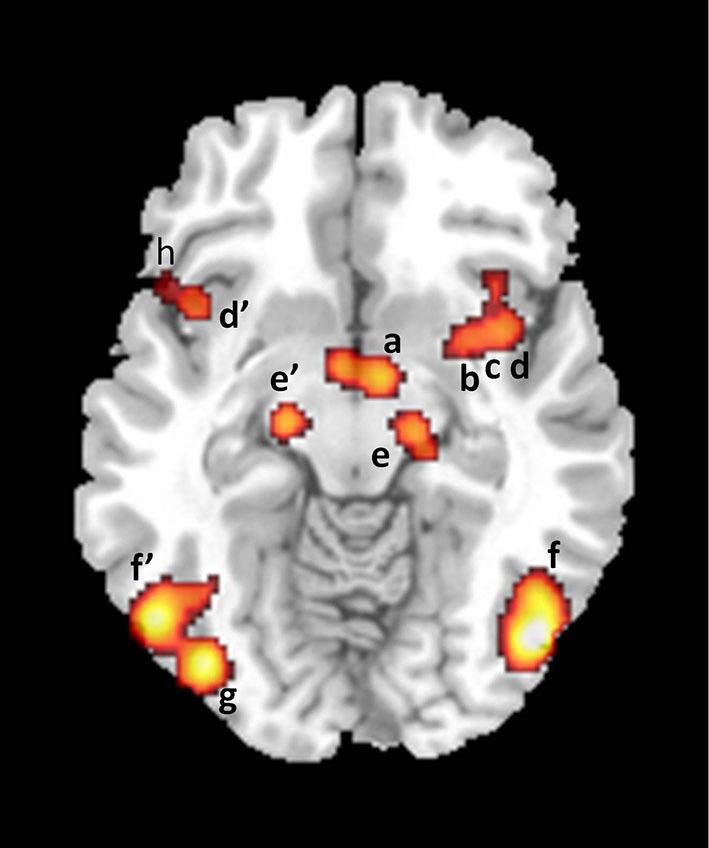
**Axial section showing activations found in a meta-analysis of 21 studies of brain responses of heterosexual men to sexual stimuli (unpublished figure from study by Stoléru et al., 2012).** Section is located 10 mm below bicommissural plane. a: hypothalamus; b: putamen; c: claustrum; d, d’: insula; e, e’: substantia nigra; f, f’: inferior temporal gyri; g: inferior occipital gyrus; h: inferior frontal gyrus. Right is to the right.

The motivational component comprises the processes that direct thoughts and/or behavior to a sexual goal, including the perceived urge to express overt sexual behavior. The motivational component includes sexual desire—but is not limited to this conscious experience. The model suggests that the anterior cingulate cortex (ACC), the hypothalamus, the nucleus accumbens and the substantia nigra (SN) are neural correlates of this component (Figures 3 and 4).

The autonomic and neuroendocrine component includes various bodily responses (e.g., genital, cardiovascular, changes in hormonal plasma levels) leading to a state of physiological readiness for sexual behavior. According to the model, the activation of the ACC (Figure 3), anterior insulae, putamens and hypothalamus (Figure 4) participate in the mediation of the autonomic/neuroendocrine responses of SA. These four components are conceived as closely coordinated. For instance, the emotional component is partly based on the perception of bodily changes generated by the autonomic component; similarly, a recent meta-analysis indicates that the right claustrum interconnects the neural networks of the psychological aspects of SA and those of its somatic processes (Poeppl et al., 2013).

## The relation between the Freudian theory of drives and the neurophenomenological model of sexual arousal

Do functional neuroimaging studies of SA confirm the Freudian theory of sexual drives? Do they simply reframe it? Or, do they invalidate it and make it obsolete?

As mentioned above, Freud (1915a) acknowledged that theories begin with concepts that are not clearly defined. This is why Freud was so cautious when he introduced the concept of sexual drives. Nobody has ever seen drives under the lens of a microscope; no radiological device has demonstrated their existence as objective entities. When Freud was writing that no science began with clear and sharply defined basic concepts, he was to introduce the concept of sexual drive, which refers to the inferred basis of a subjective experience as contrasted with an observed objective entity.

Although, drives per se are not conscious, the psychoanalytical theory of sexual drives provides a very good account of the conscious phenomenology of sexual desire: indeed, the conscious experience of sexual desire is consistent with the existence of sexual drives that exert pressure for motor expression, tend to reach an aim, make use of an object and likely have an internal bodily source. By contrast, neuroscience per se cannot provide such a phenomenological account: even if neuroscience could provide a complete and objective description of all the responses of the brain regions to VSS, that description would not convey what it is to feel sexual excitement.

We are trying here to determine whether certain features of the subjective experience derived from sexual drives have objective neural correlates. Sexual drives are the basis of conscious experiences, even if they may secondarily become repressed and unconscious. Thus, the neurophenomenological model could account for at least the conscious aspects of sexual desire derived from sexual drives. Hereunder, we examine each of the four components of the neurophenomenological model and try to indicate how it relates to the Freudian conception of sexual drives. We also consider the inhibitory aspects of the model and examine their relations to the Freudian theory regarding the repression and the inhibition of sexual drives.

Here, some terminological clarifications are in order about sexual desire, excitation and libido. By the expression desire, we refer to the felt propensity or urge or impulse to engage in sexual acts. The terms excitement, arousal and excitation encompass more diverse manifestations: they refer to a state where the subject experiences desire and/or bodily changes (genital, respiratory, etc.) and/or cognitive activity (sexual fantasies, plans of seduction, etc.) and/or emotions (joy, apprehension, tension, etc.). Regarding the term libido, “In psycho-analysis […] the term “libido” does not mean psychical energy in general but the motive force of the sexual instincts” (Freud, 1923).

### The cognitive component

The experimentally presented visual stimuli are assessed by subjects as to their sexual relevance both qualitatively (“Is this a sexual stimulus?”) and quantitatively (“How sexually arousing is this stimulus?”). Thus, the cognitive component comprises a process of appraisal through which each stimulus is categorized—or not categorized—as a sexual incentive and quantitatively evaluated as such. The target is assessed as corresponding, or not corresponding, to the category of persons to whom the subject is sexually oriented, e.g., adult women in the case of heterosexual males, and, within this preferred category the target is appraised to determine whether or not it presents certain features sexually attractive for the subject (body shape, etc.). In our proposed model, this complex analysis is conceived as performed by various brain regions, including the fusiform gyri and the orbitofrontal cortex. In other words, once the upstream visual areas have analyzed physical characteristics of the objects (gender, body shape, etc.), the assignment of sexual relevance is performed by the inferior temporal and the orbitofrontal cortices, which opens the way to motivational, emotional and bodily responses.

In the proposed model, increased attention devoted to sexually relevant targets is reflected in the activation of regions involved in sustained attention, i.e., the superior and inferior parietal lobules, which are consistently activated in functional neuroimaging studies of visually induced SA (Kühn and Gallinat, 2011; Stoléru et al., 2012; Poeppl et al., 2013; Sescousse et al., 2013).

What is the relationship between the appraisal process of the cognitive component and the object of sexual drives as a psychoanalytic concept, defined as the “thing in regard to which or through which the instinct is able to achieve its aim” (Freud, 1915a)? Such a definition implies that certain categories of “things”—usually other persons endowed with particular characteristics—are experienced by a given subject as stimulating sexual desire. In the proposed model, the inferior temporal and the orbitofrontal cortices are seen as the neural correlates of the operations through which subjects assess stimuli as corresponding, or not corresponding, to the objects of their sexual drives. Clearly, the sexual relevance of visual stimuli is not appraised by individuals as if they were blank screens or tabulae rasae; when they engage in this appraisal process, they have long-standing sexual preferences established during their development. Thus, appraisal is performed in relation to internal references, or memory traces in the language of psychoanalytic theory, which define the characteristics of the objects of sexual drives. The demonstration of hippocampal activation—a key memory area—in a meta-analysis of functional neuroimaging studies of SA is consistent with the view that appraisal is performed in relation to internal references (Poeppl et al., 2013). Thus, in the context of functional neuroimaging studies of SA, the appraisal process can be conceived as the assessment of the match between the external visual stimuli and the internal references. We propose that, while functional neuroimaging studies cannot image the objects of sexual drives, they do image the functional processes through which subjects appraise the match between visual stimuli and the internal references that define the objects of their sexual drives.

### The motivational component

Once a visual target is perceived as sexually relevant, a motivational value gets attached to it. This process makes stimuli highly salient, attractive and “wanted” (Robinson and Berridge, 1993). The motivational component is certainly the most crucial aspect of the model. Actually, the term “drive” itself refers to this motor aspect, as a drive is something that tends to make subjects move. Importantly, motivational processes are interfaced with cognitive processes. If a motivational process cannot give way to actual behavior, it will tend to trigger the emergence of representations of the behavior, i.e., motor representations. This happens in particular when actual behavior must be inhibited. Thus, although motivational and cognitive aspects are presented separately for purposes of clarity, they are closely related processes.

As is apparent from the definition above, the motivational component corresponds to two core features of drives: (i) the motor factor, i.e., the feeling of a pressure to act in a sexual manner; and (ii) the intermediate aim of drives (see above), i.e., the pattern of action that the drives strive to execute in order to reach satisfaction. As shown below, VSS induce the activation of several areas belonging to the motor system, which is consistent with the above cited Freudian assertion (“Every instinct is a piece of activity”). We now review the evidence that some activated areas are parts of the motivational component and delineate their relation with features of sexual drives.

According to the neurophenomenological model, once visual stimuli have been appraised as sexually relevant, the processing of these stimuli activates premotor areas, which might lead to overt actions if circumstances made it possible and appropriate. Within the context of a neuroimaging scanner, subjects cannot perform sexual actions so that motor processes are conceived as activating the premotor stage and the representational/cognitive aspects of motivational processes. The activation of the premotor stage could account for the observed widespread activation of the supplementary motor areas, of the ventral premotor areas, and of the region corresponding to the cingulate motor areas (Kühn and Gallinat, 2011; Stoléru et al., 2012). The activation of these premotor areas is linearly correlated with the level of perceived SA (Redouté et al., 2000). In domains other than sexuality, the activation of premotor areas has been related to conscious motor intention (Haggard, 2005).

The ventral premotor area and the supplementary motor area have distinct functions. The lateral premotor cortex—to which the ventral premotor area belongs—uses information from other cortical regions to select movements appropriate to the context of the action (Purves et al., 2001). Its neurons seem to be particularly involved in the selection of movements based on external events. In subjects presented with VSS, activation in the ventral premotor area may reflect externally triggered preparation of movements.

The medial premotor cortex, to which the supplementary motor area belongs, also mediates the selection of movements. However, this region appears to be specialized for initiating movements specified by internal rather than external cues. In contrast to lesions in the lateral premotor area, removal of the medial premotor area reduces the number of self-initiated movements a monkey makes, whereas the ability to execute movements in response to external cues remains largely intact (Shima and Tanji, 1998). Imaging studies suggest that in humans this cortical region functions in much the same way. For example, PET scans have shown that this region is activated when subjects perform motor sequences from memory (i.e., without relying on an external instruction; Grafton et al., 1998).

In keeping with the notion that activation of premotor areas are correlates of the representational/cognitive aspects of motivational processes, their activation has been interpreted as the neural correlate of sexual motor imagery reported by subjects after the presentation of VSS (Mouras et al., 2003; Stoléru et al., 2003; Moulier et al., 2006). VSS do trigger the activation of areas belonging to the neural network mediating motor imagery (inferior parietal lobules, left ventral premotor area, right and left supplementary motor areas, cerebellum). Although “sexual motor imagery” is an expression used in neuroscience, this expression refers actually to experiences that are close to, if not identical with, conscious sexual phantasies, at least to those phantasies where subjects appear as active. While the production of phantasies does not belong to the four core features of sexual drives as described by Freud, he does refer to this production in countless passages of his works (e.g., Freud, 1905, 1920, 1921).

Finally, it has been proposed that the activation of the ventral part of the lateral premotor cortex reflects the increased activity of mirror neurons (Stoléru et al., 1999; Bocher et al., 2001; Ponseti et al., 2006; Mouras et al., 2008). Mirror neurons are a particular class of visuomotor neurons, originally discovered in the monkey premotor cortex, that discharge both when a monkey performs a particular action and when he/she observes the same action performed by another monkey (Rizzolatti and Craighero, 2004). Thus, the activation of these neurons in the observer’s brain mirrors the activation of the corresponding neurons in the observed agent. The theory of the function of the mirror neurons has been expanded to the domain of emotion to account for empathy. Furthermore, we have suggested that a similar mechanism mediated by the mirror-neuron system prompts the observers of VSS to resonate with the motivational state of other individuals appearing in visual depictions of sexual interactions, with observers activating motor representations associated with the observed depictions (Mouras et al., 2008). This interpretation is reinforced by the fact that VSS also induce an activation of the inferior parietal lobule, another region that contains neurons belonging to the mirror-neuron system. These considerations may be important to explain the mechanisms of the widespread attraction to pornographic movies.

Some post-Freudian psychoanalysts, notably the Lacanian school, have elaborated the concept of identification to the desire of the Other: “The object of man’s desire, and we are not the first to say this, is essentially an object desired by someone else” (Lacan, 1953). The potential activation of the mirror-neuron system in response to VSS may thus represent neural correlates of the tendency to emulate the manifestations of sexual desire observed in others, whether directly or through films. This finding suggests the possibility that processes of identification with significant others involve not only identification with their ego or superego functions, but also with aspects of their id. Importantly, this aspect of desire is not well accounted for by the structural model of the psyche (id, ego and super-ego). As the super-ego is commonly conceived as working in contradiction to the id, e.g., maintaining our sense of morality and proscription from taboos, it does not accomodate sexual desire as an identification with aspects of the id of significant others. We propose the concept of super-id as a way to conceive that aspect of desire. This concept, grounded in functional neuroimaging studies of sexual desire, might be a way in which they could help psychoanalysis to further specify certain aspects of the theory of sexual drives.

Another area implicated in conscious motor intention and found activated in functional neuroimaging studies of SA is the posterior parietal cortex. In patients undergoing awake brain surgery, experimental stimulation of the posterior parietal cortex (Brodmann areas 39 and 40) triggered a strong intention and desire to move, e.g., stimulating right parietal regions induced the desire to move the contralateral hand, arm, or foot (Desmurget et al., 2009). Brodmann area 40 was found activated, or positively correlated with penile erection in many neuroimaging studies of SA (Kühn and Gallinat, 2011; Stoléru et al., 2012). This activation could be a correlate of the participants’ expressed desire to perform sexual actions in response to VSS, e.g., the same actions as those depicted in the clips (Redouté et al., 2000).

In the ACC, premotor areas are located in the region identified as the cognitive division (Picard and Strick, 2001). From our perspective, cingulate motor areas are particularly relevant as they may represent an interface between the limbic and the motor systems. The role of the caudal ACC in motor function is known to be similar to the role of premotor and supplementary motor area cortices (Dum, 1993). In the monkey, stimulation of the ACC elicits genital manipulation of a masturbatory character (Robinson and Mishkin, 1968). In human partial seizures with motor sexual manifestations (such as pelvic thrusting), the origin of discharge has been located in the ACC (Landré et al., 1993). The consistently observed response of the caudal ACC to VSS (Stoléru et al., 2012; Poeppl et al., 2013; Sescousse et al., 2013) is thus in keeping with the conclusion that the caudal ACC plays a crucial role in the initiation of goal-directed behaviors (Devinsky et al., 1995), which includes sexual behavior. However, in the neuroimaging paradigms used to study SA, where the urge to act conflicts with the instruction to withhold any overt behavior, there must be control mechanisms at least as powerful as the motivational processes. Activation in the caudal ACC was found in a study of a GO/NO-GO task (Kawashima et al., 1996). More generally, it is activated when there is conflict between possible responses (Carter and van Veen, 2007). According to Picard and Strick (2001), a specific part of the cingulate sulcus [located at *y* = 24 mm ± 7 mm (mean ± SD, in Talairach coordinates)] appears to be involved in conflict monitoring. Interestingly, several studies on SA have reported activation in this area (Stoléru et al., 1999; Arnow et al., 2002; Karama et al., 2002; Moulier et al., 2006; Safron et al., 2007; Sundaram et al., 2010), which suggests that this activation may correspond to conflict monitoring. Accordingly, we propose that the activation of the caudal ACC in response to VSS results from conflicting inputs to this area: inputs of the GO type, correlated with the perceived urge to enact SA, and inputs of the NO-GO type, correlated with the perceived need to withhold any overt sexual behavior in the current circumstances. Both types of inputs would be associated with activation, because it is the local synaptic activity that is energy consuming.

On the one hand, the neurobiological account of SA proposes a conflict between two kinds of inputs; on the other hand, an essential characteristic of sexual excitement is a “peculiar feeling of tension of an extremely compelling character”(Freud, 1905). The activation of the above described region of the ACC may thus represent a neural correlate of the experience of sexual tension.

While cortical regions are associated with the motivational component, this component also includes subcortical areas. The latter areas are important as they may lead to the causation of sexual desire and to the sources of sexual drives, better than do cortical areas. In animals, the medial preoptic area has repeatedly been implicated in sexual motivation or sexual behavior in males (e.g., Swanson, 2000; Hull and Dominguez, 2006; Balthazart and Ball, 2007) whereas the ventromedial nucleus of the hypothalamus is important for the expression of female sexual behavior, in particular, the lordosis reflex (Pfaff, 1999). In an experiment performed in monkeys (Oomura et al., 1988), the rate of discharge of neurons in the medial preoptic area of an experimental male increased when he viewed a receptive female and performed button presses resulting in bringing the female close to him. In humans also, areas activated in response to VSS include the medial preoptic area (Stoléru et al., 2012; Sescousse et al., 2013).

In response to VSS, the ventral striatum (nucleus accumbens) was found activated in about a quarter of functional neuroimaging studies of SA (27.0%), a rather low percentage given its widely reported involvement in animal studies of sexual motivation and in studies of other motivational processes. Nevertheless, this region was found in a meta-analysis of brain responses to VSS (Sescousse et al., 2013). In functional neuroimaging studies of SA, the level of activation in the ventral striatum was also found correlated with the degree of perceived SA (Redouté et al., 2000; Walter et al., 2008). Two main interpretations of this activation have been proposed. Firstly, it has been conceived as the neural representation of received reward, as VSS are experienced as rewards in themselves (Sabatinelli et al., 2007; Walter et al., 2008; Sescousse et al., 2010). Secondly, this activation has been related to the incentive motivational aspects of VSS and to anticipated reward (Ponseti et al., 2006). But what exactly does “reward anticipation” mean in terms of subjective experience? It may mean the cognitive operation whereby a subject knows that a specific rewarding outcome is going to occur. It may also denote the craving or desire associated with such knowledge.

By contrast, the head of the caudate nucleus (dorsal striatum) has been found activated much more consistently (Stoléru et al., 2012; Poeppl et al., 2013; Sescousse et al., 2013; Figure 3). This region belongs to the nigrostriatal pathway, a dopaminergic (DA) pathway connecting the SN with the striatum (see below). As part of a system called the basal ganglia motor loop, this pathway is involved in the production of motor behavior. We shall also describe in more detail how the head of the caudate could also play a role in inhibiting the expression of overt sexual behavior, once SA has been induced (see section Inhibition of Overt Behavioral Expression). As in the case of the ACC, the same structure could contain both neurons involved in triggering sexual behavior and neurons that tend to inhibit its expression.

What is the relationship between, on the one hand, the motivational component just reviewed and, on the other hand, the pressure factor as a core feature of sexual drives? In domains other than sexuality, the brain areas of the motivational component are known to be correlates of intentional behavior and/or motor imagery. Some of these areas are cortical. At this “higher level” of motivational processes, it is likely that the motor factor (i.e., the urge to move) and the goal of actions (conceived as the conscious imagery of specific patterns of action) are integrated into the desire to perform specific patterns of action. In other words, we propose that the conscious imagery of specific actions and the urge to perform them are correlated with the activation of the cortical regions of the motivational component, with an integration between the urge felt and the representation of the pattern of actions. Thus, what Freud described as two distinct aspects of sexual drives—the motor factor and the intermediate aim of drives—may be integrated into a single entity, namely the pressure to perform specific actions, and correlated with the activation of a single neural network.

It has recently been proposed to distinguish brain regions involved in the representation of pleasure and those involved in the causation of pleasure (Berridge and Kringelbach, 2013). The same distinction between causation and representation could be useful in the case of motivation. Both animal and human studies indicate that the DA neurons located in brainstem nuclei—mainly in the ventral tegmental area (VTA) and the SN—are key elements of motivational processes in general, i.e., not specifically of sexual motivational processes. These general motivational processes rely on DA pathways that appear essential in the causation of sexual motivation. Hereunder, we briefly describe the main DA pathways, and point out their possible involvement in SA and relation with sexual drives as conceived by Freud.

In the nigrostriatal pathway, perikarya arise in the SN and project to the caudate nucleus and the putamen, known collectively as the striatum; nigrostriatal DA neurons are involved in the initiation and execution of copulatory movements (Hull et al., 2004). The bilateral activation of the SN and of the caudate nucleus is a consistent finding across functional neuroimaging studies of SA (Stoléru et al., 2012). The activation of this pathway could be a correlate of the pressure factor of sexual drives, i.e., of the urge to perform motor acts in response to sexually relevant stimuli.

In the mesolimbic pathway, perikarya located in the VTA project diffusely to limbic and cortical structures, including the nucleus accumbens, the amygdala, and the ACC. Various theorists have proposed a motivational interpretation of mesolimbic DA pathway functioning (Alcaro et al., 2007). In the framework of incentive motivation theory (Robinson and Berridge, 1993), “incentive salience” is a psychological process that transforms the perception of stimuli, imbuing them with salience, making them attractive, “wanted”, stimuli. In this theory, the mesolimbic dopamine projections attribute incentive salience to the perception and mental representation of events associated with activation of this pathway. Has the mesolimbic pathway been found activated in neuroimaging studies of SA? The activation of the VTA has been reported in some studies, but not consistently. By contrast, more consistent findings have been obtained regarding the involvement of the VTA in rodents’ sexual behavior (Baskerville and Douglas, 2008). VTA activation has been noted, however, in several studies of romantic love (Cacioppo et al., 2012) and in a study of the neural correlates of orgasm (Holstege et al., 2003). While the origin of the pathway, i.e., the VTA, has relatively rarely been found activated, two of its target areas have been consistently found activated: the ACC and the amygdalas (Figures 2, 3 and 4). However, although amygdalar activation has been related to motivational processes (Hamann et al., 2004), according to current interpretations (Murray, 2007; Walter et al., 2008) the amygdala would process the emotional aspects rather than the motivational relevance of VSS (see below: “The emotional component”).

Like the mesolimbic pathway, the mesocortical pathway originates in the VTA but terminates in the cortex, in particular in the frontal lobe. A recent study supports the view that activation of the VTA and SN regions induced through sexual or romantic imagery could result in widespread cortical activation (Sulzer et al., 2013). Participants were trained to control the level of activation in a region encompassing the SN and the VTA. To achieve this control, real-time fMRI provided subjects with a visual feedback of their SN/VTA activity while they were imagining rewarding scenes—actually sexual or romantic imagery. To examine the effect of neurofeedback, subjects were assigned to either a group receiving feedback directly proportional (veridical feedback) or a group receiving inversely proportional (inverted feedback) to SN/VTA activity. In both groups, sexual or romantic imagery was associated with activation of the SN/VTA region and with activation in frontal, parietal, temporal and occipital cortices. In both groups, analysis showed increased connectivity of the SN/VTA region with the dorsal and ventral striatum; surprisingly, functional connectivity between the SN/VTA region and widespread cortical areas was demonstrated only in the inverted feedback group. If the involvement of the mesocortical pathway in SA is confirmed, it could provide an anatomical basis for the widespread cortical activation associated with SA. This cortical activation could in turn help understand the generation of sexual thoughts and imagery associated with sexual motivation.

Interestingly, a parallel can be drawn between the concept of libido and the idea that sexual thoughts are fueled by mesocortical DA projections. Just as the libido can become attached to different objects (across various persons and during one person’s life history), it is easily conceivable that mesocortical projections establish synapses wih various cortical networks (across individuals or during one individual’s history) giving rise to various object representations associated with sexual desire. Mesocortical projections might then be the support of libidinal cathexis. Similarly, sublimation—directing some proportion of libido on to cultural aims—could be understood as the establishment of new synapses between mesocortical axons and neurons involved in intellectual or artistic cognitive processes. Furthermore, fixations of the libido could be better understood through the concept of long-term potentiation of synapses of mesocortical and mesolimbic neurons. Finally, the idea that libido is a “quantitatively variable force” (Freud, 1905) could be related to the width of arborization and rate of discharge of mesocortical and mesolimbic neurons. Conversely, irrational and primary process thoughts might depend on the impact of DA innervation of cortical networks.

Thus, functional neuroimaging studies of SA suggest that there exists a hierarchy of neural structures and processes whose activation correlate with the experience of the motor factor of drives and the associated motor imagery. The prominent role of the DA pathways in motivation suggests they may be—to use Freud’s terms—an answer biology has returned to the questions psychoanalysis has put to it regarding the source of sexual drives, seen by Freud as “the most obscure part of the psychoanalytic theory of drives” (Freud, 1920). However, the DA pathways subserve not only sexual motivation but also other types of motivation (including eating and drinking). The major difference between the Freudian and the neurophysiological views regarding the source of sexual drives is that psychoanalysis locates these sources in peripheral organs, with sexual excitations arising in certain parts of the body. By contrast, neurophysiology suggests that the source of drives could lie in the brain itself. In the neurophenomenological model, excitations originating in the body do not appear to be necessary for SA to occur, at least in the case of VSS and in adult subjects.

However, the neurophenomenological model may be constrained by its reliance on studies based for their great majority on VSS, which are external stimuli. In addition, regarding the somatic source of sexual drives, it is essential to note that testosterone, secreted by the testicles, has a major impact on sexual motivation. One of the multiple sites of action of testosterone in the brain is the medial preoptic area. In male rats, testosterone increases both basal and female-stimulated dopamine release in the medial preoptic area (Hull and Dominguez, 2006), which possibly enhances sexual motivation. However, Freud’s model of the source of sexual drives as a “process of excitation occurring in an organ” and the hormonally-mediated mechanism just mentioned are different because in the latter mechanism the process of excitation lies in the brain target of testosterone, not in the testicles. Freud himself was aware of emerging knowledge on molecules that would later be called sexual hormones: “It seems probable, then, that special chemical substances are produced in the interstitial portion of the sex-glands; these are then taken up in the blood stream and cause particular parts of the central nervous system to be charged with sexual tension”. He acknowledged the difficulty to integrate his view of erotogenic zones and emerging knowledge on substances produced by gonads: “The question of how sexual excitation arises from the stimulation of erotogenic zones, when the central apparatus has been previously charged” could not be treated, “even hypothetically, in the present state of our knowledge” (Freud, 1905). Moreover, Freud was well aware that in many cases sexual excitement could occur in the absence of sex glands, which showed that testicles cannot be considered as the sole source of sexual drives.

### The emotional component

From a phenomenological standpoint, the emotional component includes the specific hedonic quality of SA, i.e., the pleasure associated with rising arousal and with the perception of specific bodily changes, such as penile erection. In our functional neuroimaging studies, the highest ratings for pleasure were associated with the presentation of the most sexually arousing pictures (Moulier et al., 2006; Mouras et al., 2008). Other emotional responses, such as sexual tension or disgust, may occur as part of SA. In the proposed model, the activations of the uppermost part of the primary somatosensory cortex—that receives inputs from the external genitalia—, of the left secondary somatosensory cortex, the amygdalae, and the insulae are conceived as neural correlates of the emotional component (Figure 2)

The prevailing interpretation of the activation of the amygdalae is that they receive multimodal sensory inputs and participate in the evaluation of the emotional content of the complex perceptual information associated with VSS (Ferretti et al., 2005). Then, they relay processed information to the ventral striatum, hypothalamus, autonomic brainstem areas, and the prefrontal cortex.

The level of activation of the insulae was found correlated with the level of markers of SA—penile tumescence or perceived SA (Kühn and Gallinat, 2011; Poeppl et al., 2013). Given the involvement of the insulae in visceral sensory processing (Craig, 2003), the correlation between their level of activation and the penile response may reflect the role of the insulae in the perceptual processing of penile inputs (Moulier et al., 2006) and possibly in the awareness of erection, which would be akin to the insular function of interoceptive awareness (Craig, 2003). This role of the insulae in the sensory processing of penile inputs is consistent with the report that manual stimulation of the penis strongly activates the right posterior insula (Georgiadis and Holstege, 2005).

In the Freudian view of sexual excitement, the emphasis is laid on the concept of sexual tension and on its opposite, sexual satisfaction. The goal of sexual behavior is to reach satisfaction through the elimination of that tension, as satisfaction can only be obtained by removing the state of stimulation at the source of the instinct. Importantly, preliminary sexual activities are themselves accompanied by pleasure and on the other hand they intensify both the excitement and the tension. For instance, “A certain amount of touching is indispensable (at all events among human beings) before the normal sexual aim can be attained. And everyone knows what a source of pleasure on the one hand and what an influx of fresh excitation on the other is afforded by tactile sensations of the skin of the sexual object” (Freud, 1905). In an incremental cycle, such fresh excitation leads subjects to further sexual activities that themselves generate increasing pleasure, tension and excitation. As noted in the first section, Freud recognized this peculiar mixture of tension (unpleasure) and pleasure.

Do neuroscientific findings account for this emotional duality of SA? This duality is reminiscent of research conducted in rodents to disentangle neural circuits involved in pleasure (“liking”) and in motivation (“wanting”). These studies have focused mainly on feeding, but also on sex behavior. They show that mesolimbic dopamine, which was widely considered the brain neurotransmitter candidate for pleasure two decades ago, turns out not to cause pleasure or “liking”. Rather, dopamine mediates more selectively a motivational process of incentive salience, which is a mechanism for “wanting” rewards but not for “liking” them (Berridge and Kringelbach, 2013). By contrast, opioid and endocannabinoid neurotransmitters do more effectively generate intense pleasures—but only within so-called “hedonic hotspots”. For example, stimulation of mu opioid receptors within a hotspot of the nucleus accumbens (a hotspot localized in the rostrodorsal quadrant of the medial shell of the nucleus accumbens), or in another hotspot of the ventral pallidum (in the posterior half of ventral pallidum), more than doubles the intensity of “liking” reactions elicited by sweetness. But the same stimulation of mu opioid receptors elsewhere in the remaining 90% of the nucleus accumbens generates only “wanting” without enhancing “liking”—much like dopamine. Thus, these studies indicate that affects of pleasure and motivational responses are related to specific and distinct subcortical structures. Each hotspot is only a cubic millimeter in rats; then, a human hotspot equivalent should be approximately a cubic centimeter, if scaled to whole-brain size. Importantly, the posterior ventral pallidum was found to be activated in a meta-analysis where no restriction was imposed on the size of clusters of activation concordant across neuroimaging studies (Stoléru et al., 2012; *x, y, z*: −24, −10, 0, MNI coordinates, unpublished finding). The right nucleus accumbens was also found activated in one of the meta-analyses (Sescousse et al., 2013).

Although orgasm is an essential aspect of the emotional component, we shall not attempt to relate this experience to its potential neural correlates. One reason is that orgasm is not an essential part of Freud’s theory of sexual drives. In addition, functional neuroimaging studies of the neural correlates of orgasm have yet to overcome many difficult methodological problems which have prevented the emergence of consistent results. However, clinical neurology has shown that orgasm can occur in female and male patients as a consequence of lesions in the temporal lobe and/or temporal lobe epilepsy. As an example, a 44-year-old woman experienced for a period of 3 years recurrent episodes in which she would suddenly become aware of a feeling indistinguishable from an orgasm (Reading and Will, 1997). An electroencephalogram showed a right frontotemporal epileptic focus. Radiological examinations revealed a large vascular abnormality in the right temporal lobe. Antiepileptic drug therapy put an end to the occurrence of seizures and to accompanying orgasms. Although rare, such cases have a great theoretical importance because they show that orgasm may occur both in women and men in the absence of any genital stimulation. Thus, a pattern of brain activation, independent of the stimulation of any erotogenic zone, can result in orgasm.

### The autonomic and neuroendocrine component

The autonomic and neuroendocrine component consists in the bodily changes that result from sexual stimulation, including genital, respiratory and cardiovascular responses. These changes also include hormonal responses, such as a rise in blood testosterone levels, which has been recorded in men and in all other mammals so far studied, from rats (Bonilla-Jaime et al., 2006) to bulls (Katongole et al., 1971). These autonomic and hormonal changes are understood as preparing the body for copulation. Furthermore, some of these changes, e.g., genital sensations, further contribute to SA, thus generating a positive feedback loop. According to the model, activation in the ACC, anterior insulae, putamens and hypothalamus participates in the mediation of the autonomic and neuroendocrine responses of SA.

This set of bodily changes is distinct from what Freud called the somatic source of sexual drives. They are consequences of the brain processing of sexual stimuli, not the cause of SA. However, in particular in the case of erection, the perception of bodily changes is part of a positive feedback loop where arousal contributes to further arousal. As seen above, midbrain regions (SN, VTA), which provide the origins of DA pathways, could play an essential role in the causation of sexual motivation. In that case, the source would be located in the central nervous system rather than in peripheral organs. Of course, this central mechanism would not eliminate the role of external stimuli and peripheral receptors, but it would mean that these inputs, in and by themselves, are not the source of drives. In a brief passage, Freud acknowledged the existence of a centrally conditioned sensation of sexual stimulation: “The state of being in need of a repetition of the satisfaction reveals itself in two ways: by a peculiar feeling of tension, possessing, rather, the character of unpleasure, and by a sensation of itching or stimulation which is centrally conditioned and projected on to the peripheral erotogenic zone” (Freud, 1905). This passage sounds like a forerunner of the “as if body loop” of the theory of somatic markers (Damasio, 1996) where reasoning and decision-making about a factual situation is influenced by a concomitant “somatosensory image” that defines an emotional response. The somatosensory image is related to a somatosensory activation that can occur in one of two ways: (i) via a “body loop”, in which emotion can be evoked by actual changes in the body that are then projected to the somatosensory cortices or (ii) via an “as if body loop”, in which top-down signaling from regions such as orbitofrontal cortex and amygdala drives activity in somatosensory maps from within the brain, independently of actual body signaling. Similarly, in the domain of sexual desire, actual body loops or “as if body loops” may be activated so that the subjective perception of a given episode of arousal does not remain undifferentiated but is imbued with a specifically sexual character. In that case, the body would not be the source of sexual excitation but would function as a marker of the sexual character of arousal.

If, as posited by Freud, sexual drives are exerting a constant pressure to get expressed behaviorally, and if the SN/VTA is a neural correlate of the source of drives, the SN/VTA should contain neurons which are firing tonically, except after satisfaction has been reached. Indeed, *in vivo* rodent and monkey VTA and SN DA neurons display basal tonic firing rates of up to 20 Hz (Radulescu, 2010). This view predicts that these neurons should show decreased rates of firing during the sexual refractory period. Unfortunately, the neural correlates of that period are not well known. In the only fMRI study of sexual satiety (Mallick et al., 2007), the amygdalas, temporal lobes and septal area were more active 3 min after ejaculation than after 30 min. As the latter duration was considered as longer than the postejaculatory refractory period, the above-mentioned activations might be neural correlates of the refractory period, leading to hypothesize that these areas might exert inhibition on SN/VTA neurons.

Our review of the last two components of the model leads us to the following questions: Is it tenable that “the sources of sexual drives are somatic processes which occur in an organ or part of the body”—in particular in erotogenic zones (Freud, 1915a)? What is an erotogenic zone in the light of functional neuroimaging studies of SA? Can we maintain that the pleasure derived from the stimulation of an erotogenic zone such as the clitoris or the penis has its source in these organs, or should we rather consider that genital inputs reach brain regions that generate pleasurable sensations? To answer these questions, it is important to consider the brain targets of genital afferents. Using a trans-synaptic viral tracer in order to provide a full map of the functional genitosensory pathway in rats, Normandin and Murphy (2011) have observed viral labeling in the paraventricular nucleus of the hypothalamus in both sexes. Viral labeling was also observed in males, but not in females, in the SN, VTA, thalamus, central amygdala, anterior insular cortex and in the ventro-orbital cortex. All these regions demonstrated in the rat have been found activated in meta-analyses of the neural correlates of human SA in studies predominantly based on visual—not tactile—stimuli (Stoléru et al., 2012). Thus, while tactile genital stimuli converge on areas similar to those that process VSS, genital stimuli may not have a special function as sources of sexual drives. In addition, in humans, as shown by a meta-analysis of functional neuroimaging studies of subjective pleasantness (not limited to sexuality), two of the brain areas found in the rat study, the medial orbitofrontal cortex and the left thalamus, were among the neural correlates of pleasure (Kühn and Gallinat, 2012). Thus, while pleasurable sensations seem to arise in the genital organs, actually the pleasurable character is probably the result of central stimulation triggered by inputs from the genitals.

The above reasoning refers to the hedonic, “liking”, aspects of genital sensations. Should the same reasoning be applied to the motivational, “wanting”, aspect of genital inputs? The answer is likely positive as experiences of centrally mediated genital pleasure are learned and motivate future sexual pleasure seeking. In addition, genital organs may function as somatic markers of sexual motivation, much as other body parts do in the case of emotional responses. Thus, genital sensations may inform subjects on the sexual nature of their motivational state, just as acceleration of heartbeat help them to realize and label their feelings of fear, tension in the jaws their feelings of anger, and stomach sensations their feelings of hunger. However, these body parts are not, in and by themselves, the sources of those emotional or motivational states. In summary, while genital organs with an adequate innervation are important components of sexual pleasure and desire, the final generation of pleasurable sensations and sexual motivation depends on their target brain regions.

### The inhibitory components

The proposed model comprises three distinct inhibitory components.

#### Tonic inhibition from temporal cortical areas

We first postulated the existence of a tonic inhibitory component after noting that SA was related to a decreased activation in some regions within the right and left lateral temporal cortices (Brodmann areas 20, 21, 22, 39) and in the right medial orbitofrontal cortex (MOFC; Redouté et al., 2000). The model holds that these regions exert a continuous inhibition of areas implicated above in the development of SA. For SA to unfold, there must be a deactivation of these inhibitory structures. This model is consistent with the hypersexual syndromes observed in patients whose lateral temporal regions are injured, either by pathological processes such as tumors (Burns and Swerdlow, 2003) or after experimental or therapeutic surgery [Klüver-Bucy syndrome in monkeys (Klüver and Bucy, 1939); and its counterpart in humans (Terzian and Dalle Ore, 1955)].

How does this first component of inhibitory processes relate to the Freudian theory of sexual drives? Interestingly, regions proposed to mediate tonic inhibition of SA have been found activated in tasks involving “moral feelings”, such as guilt and embarrassment (Berthoz et al., 2002; Takahashi et al., 2004). These feelings are described as barriers to the sexual drives, erected during the latency period (Freud, 1905). Psychoanalysis has put a great emphasis on the repression of the representational aspect of sexual drives, whereby particular thoughts are prevented to become conscious. However, Freud also described the repression of what he called the “charge of affect” attached to sexual drives (Freud, 1915b). It is this “charge of affect” that tonic inhibition is conceived as countering.

It is important to distinguish tonic inhibition, which is temporary and reversible, from the concept of repression, which is sustained and not easily reversible. While in the neurophenomenological model sexual inhibition can alternate with SA in a matter of a few minutes, repression is described by psychoanalysis as an enduring process, which forms the basis of permanent characteristics of the sexual functioning of individuals. Thus, if psychoanalysis is to be compatible with findings from functional neuroimaging studies, it must account not only for permanent repression of sexual drives, but also for their reversible and temporary inhibition. Regarding the term “inhibition”, Freud uses it mainly in relation to ego functions rather than in relation to drives. When he does use this term in relation to drives, inhibition refers again to an enduring process: “[after the repression that takes place around 5 years of age] The child still remains tied to his parents, but by instincts which must be described as being “inhibited in their aim [zielgehemmte]”. The emotions which he feels henceforward towards these objects of his love are characterized as tender. It is well known that the earlier sensual tendencies remain more or less strongly preserved in the unconscious, so that in a certain sense the whole of the original current continues to exist” (Freud, 1921). The proposed model suggests to distinguish enduring inhibitions (those that Freud had in view and that are described in the next inhibitory component) from tonic—but temporary—inhibitions, dictated by external circumstances.

#### Devaluation of sexual stimuli

The existence of a process of devaluation of the erotic character of the visual targets was initially suggested by a conspicuous and surprising neural response of patients with hypoactive sexual desire disorder: in only one brain area, the MOFC, responses to VSS were higher than in healthy controls (Stoléru et al., 2003). Specifically, there was an abnormally maintained activity in the patients’ left gyrus rectus (a part of the MOFC), which contrasted with a deactivation in controls. Clinical observations are helpful to understand the functional meaning of this finding. Patients with lesions in the orbitofrontal cortex, in particular in the MOFC (Damasio et al., 1994), are often tactless, lacking in social restraints, and presenting with excessive pleasure-seeking behaviors, especially in the sexual domain (Blumer and Benson, 1975; Miller et al., 1986). Thus, the functional deactivation of the MOFC seen in healthy subjects upon VSS presentation suggests a release of SA from the inhibitory control of MOFC, while in patients with hypoactive sexual desire disorder the maintained activity of this region is consistent with a maintained inhibition of the unfolding of SA. Patients with this disorder said on debriefing that, when presented with VSS, they felt “indifferent”, “as if I had been watching a catalogue” (Stoléru et al., 2003). Importantly, studies performed on nonsexual motivated behaviors have shown that the MOFC codes for the lack of reward expected in a given situation; this is the case in animal experiments using the extinction procedure, where the discharge of MOFC neurons was recorded when reward was withheld (Rosenkilde et al., 1981). Therefore, in animals, lesions of the MOFC result in a marked tendency toward continued response during extinction paradigms (Butter, 1969). Conversely, a role has been demonstrated for neurons of the ventromedial prefrontal cortex—a region to which the MOFC belongs—in the memory for extinction (Quirk et al., 2000). Although the outcome of the extinction paradigm and the low desire response of patients with hypoactive sexual desire disorder are clearly different situations, we have suggested that they share an important feature, i.e., in both cases the motivational significance of a stimulus has been devalued and a motor response is no longer warranted. In these patients, the maintained activity of the MOFC neurons might thus be coding for the learned suppression of anticipated reward in situations of sexual stimulation and/or for a higher restraint of downstream motivational processes. Thus, while the MOFC presents a deactivation in responses to VSS, the more lateral orbitofrontal cortex shows activation. In the model described above, these contrasting responses correspond to distinct functions.

Is there any concept in the psychoanalytical theory of sexual drives that is related to the mechanism of devaluation? Actually, the concept of sexual drives as inhibited in their aim (see above) is much closer in two respects to this second inhibitory mechanism than to the first (tonic inhibition from temporal areas). Firstly, when a sexual drive attached to a given object is inhibited in its aim, the object loses its sexual attraction. Secondly, inhibition of sexual drives in their aims is an enduring phenomenon just as the devaluation of sexual objects, at least in patients with hypoactive sexual desire disorder, is an enduring process that is very difficult to reverse by psychotherapy.

Is there another form of devaluation of sexual stimuli that would be transient, reversible and found in normal subjects? That the healthy controls present a deactivation in the MOFC in response to VSS (Redouté et al., 2000) suggests that sexual quiescence includes a high activity of the MOFC, maintaining a low level of desirability of potential sexual stimuli. If such reversible forms of devaluation exist, the process of devaluation shown by neuroimaging studies in healthy subjects and the process of inhibition of sexual drives in their aims delineated by Freud operate on different time scales: whereas devaluation can be temporary (in healthy subjects) or enduring (in patients), inhibition of sexual drives in their aims operates generally on a long time scale. The process of devaluation is also different from the inhibition of sexual drives in their aims for another reason: according to Freud, inhibition of sexual drives in their aims enables individuals to establish positive relationships that are not directly sexual, but whose roots are to be found in sexual drives inhibited in their aims: “All the ties upon which a group depends are of the character of instincts that are inhibited in their aims” (Freud, 1921). By contrast, enduring devaluation described in the proposed model is associated with pathological low desire and with no constructive relationship.

#### Inhibition of overt behavioral expression

Based on a model of basal ganglia function (Rolls, 1999), we have proposed that once SA has been induced, the activation of the right caudate nucleus reflects inhibitory processes controlling the overt behavioral expression of SA (Redouté et al., 2000). In this model, once cortical neurons have decoded the motivational significance of stimuli, their reward-related signals should not be interfaced directly with motor behavior. Instead, the signals enter an arbitration mechanism which takes into account the cost of obtaining reward in the present circumstances. It has been proposed that the caudate nucleus participates in this function (Rolls, 1999). The model is supported by clinical evidence, as hypersexuality has been reported in patients with lesions circumscribed to the head of the caudate nuclei (Richfield et al., 1987). It is also consistent with evidence from neuroimaging studies in domains other than sexual behavior, i.e., (i) the activation of the putamen and/or the caudate nucleus in paradigms where the need for a motor response is conflicting with the need to withhold it (Pardo et al., 1990); and (ii) the activation of the head of the caudate nucleus upon volitional tic suppression in Tourette Syndrome (Peterson et al., 1998). In addition to the inhibitory function of the caudate, we have seen above that part of the activation of the caudal ACC has been interpreted as the correlate of inputs of the NO-GO type. Is there any counterpart in Freudian theory for this kind of inhibitory mechanism? This seems not to be the case as Freud described defense mechanisms against sexual drives mainly as enduring characteristics of ego functioning.

## Limitations and future directions of research

Firstly, functional neuroimaging studies of SA have all been conducted in adult subjects. Thus, they cannot throw any light on the development of sexual drives which according to Freud starts in early childhood. Secondly, given that the criteria of SA used in most neuroimaging studies are conscious ratings, these studies cannot investigate the unconscious aspects of sexual drives which are also a major aspect of the psychoanalytic theory. To investigate these unconscious aspects, one could try to use the few functional neuroimaging studies where the presentation of subliminal VSS has been found to induce activation in only some of the regions mediating SA (e.g., Gillath and Canterberry, 2012). However, such an approach would be misleading: whereas subliminal VSS induce lower arousal, unconscious sexual drives are not described as weaker than their conscious counterparts.

Thirdly, almost all neuroimaging studies have used external stimuli. Therefore, their results may not be completely generalizable to the case where SA is triggered by internal stimuli. Future studies could test whether these results hold when SA is elicited by internal imagery. Fourthly, although studies in heterosexual women, gay men and lesbians have yielded results similar to those found in heterosexual men (Stoléru et al., 2012), the neurophenomenological model has mostly been based on studies conducted in heterosexual men. Fifthly, from a technical point of view, functional neuroimaging techniques have limited** spatial and temporal resolutions so that some neural correlates may escape detection or be identified in an imprecise way; for instance, it is difficult to identify which specific hypothalamic nuclei have been activated. Finally, the identification of regions that make up each of the four neural components of the model should be confirmed through studies aimed at demonstrating functional connectivity of these regions in response to sexual stimuli.

We have presented above a neurophenomenological model of SA that has emerged from functional neuroimaging studies. Clearly, other methodological approaches provide complementary perspectives that address other levels than those of the model and can also be put in relation with the Freudian theory of sexual drives. This is the case for genetic, neurochemical, and endocrine approaches (Pfaff and Fisher, 2012).

## Conclusions

Let us return to our initial questions: Are the results of functional neuroimaging experiments consistent with the Freudian model of sexual drives? Can modern studies help psychoanalysis to reformulate certain aspects of this model? In spite of the above-mentioned limitations, functional neuroimaging studies of SA have thrown a new light on the four fundamental characteristics of sexual drives by identifying their potential neural correlates. The neurophenomenological model based on these studies includes: (i) brain areas involved in assessing visual targets as congruent, or not, with the objects of sexual drives; (ii) brain areas related to the motor factor of drives and to their aims, the aims being understood here as specific action patterns; (iii) neural structures that may contribute to the source of sexual drives; and (iv) neural correlates of emotions involved in SA, in particular pleasure and tension. In addition, functional neuroimaging sudies demonstrate the neural correlates of the autonomic and neuroendocrine aspects of SA. Finally, neuroimaging studies have begun to identify the neural correlates of three barriers to the inappropriate expression of drives: moral feelings, temporary or enduring devaluation of sexual targets and refraining from sexual acts once SA has occurred.

The main difference emerging between the functional neuroimaging perspective and the Freudian theory relates to the source of drives. At least in adult subjects, the process of excitation occurring in a peripheral organ does not seem to be an indispensable part of the determinants of SA. It is rather the central processing of sensory stimuli that gives them their sexually arousing and sexually pleasurable character. Thus, as predicted by Freud, biology, here represented by functional neuroimaging studies, may have thrown some light on “the most obscure part of the psychoanalytic theory of drives” and begun to return some answers to the questions psychoanalysis has put to it (Freud, 1920).

## Conflict of interest statement

The author declares that the research was conducted in the absence of any commercial or financial relationships that could be construed as a potential conflict of interest.

## Footnotes

^1^Our use of the expressions “sexual excitement” in connection with Freud’s theory and “sexual arousal” in connection with the neurophenomenological model aims to be consistent with the original papers, and is not meant to refer to different phenomena.
